# Increasing flexibility in vulnerable power grids using electrochemical storage

**DOI:** 10.1016/j.heliyon.2024.e35710

**Published:** 2024-08-06

**Authors:** Gustavo Adolfo Gómez-Ramírez, Luis García-Santander, Markel Zubiaga Lazkano, Carlos Meza

**Affiliations:** a Escuela de Ingeniería Electromecánica, Intituto Tecnológico de Costa Rica, Cartago, 159-7050, Costa Rica; b Departamento de Ingeniería Eléctrica, Universidad de Concepción, Chile, Concepción, 4030000, Chile; c Departamento de Tecnología Electrónica, Universidad del País Vasco / Euskal Herriko Unibertsitatea L., Eibar, 48940 Leioa (Bizkaia), Spain; d Department of Electrical, Mechanical and Industrial Engineering, Anhalt University of Applied Sciences, Köthen, 06366, Germany

**Keywords:** Energy storage systems, Hosting capacity enhancement, Load management, Flexibility of power transfer, Power system planning

## Abstract

Developing a reliable power grid and investing in non-conventional renewable energy resources pose problems for low- and medium-income countries. Frequently, maintaining a robust power grid infrastructure can present challenges in terms of reliability, resilience, and flexibility. This article presents a methodology for improving power flexibility in susceptible power systems through the utilization of Battery Energy Storage Systems (BESS). The methodology entails the examination of power stability, operating conditions, and security criteria in order to identify suitable locations for storage allocation. A study was conducted utilizing the Electrical Transient and Analysis Program (ETAP®) software to simulate the Central American power transmission grid. The results of the study indicate that including storage systems to offer virtual inertia and backup during emergency situations is a recommended strategy for mitigating potential challenges. The study suggests that applying specific criteria for allocation and sizing at critical points in sensitive systems can enhance power transfer flexibility, eliminating potential constraints. The Central American electrical Power System, which faces power transfer limitations, is well-suited for BESS. In severe contingencies, such as when the system frequency drops to 58.75 Hz and power transfer between Mexico and Central America exceeds 300 MW with voltage levels below 0.97 pu, BESS can help mitigate these issues. The solution involves deploying BESS both centrally and distributively. Results show decreased instability, with power increases not exceeding 300 MW for more than 11 study cycles in all scenarios. The approach includes a BESS with an installed capacity of 1,060 MWh/160 MW and a virtual inertia of H=6s.

## 1 Introduction

Energy Storage Systems (ESS) perform an important role in the process of transitioning electrical networks to renewable sources of energy. The application of it has significantly evolved due to the current study, which has introduced novel opportunities for its employment. The management capabilities of energy management are essential for numerous applications, particularly for increasing the capacities of existing electrical generation resources or replacing them.

Due to the power grids are inherently heterogeneous and, if not developed in a planned manner, can lead to significant power imbalances that can affect their performance, this is even more severe in power grids with low-inertia generation power plants. Photovoltaic (PV) based distributed power generation can make imbalance problems more severe due to its intrinsic variability. Use of technologies such as PV and wind in combination with ESS (especially electro-chemical) may displace non-renewable resource options in transmission and distribution grids (T&D) and could also provide additional ancillary services to support their operation and stability.

The authors in [1] analyze the contribution of flexibility renewable energy facilities, which include wind farms, biowaste units, and storage devices for hydrogen, thermal energy, and compressed air, in the energy market using an equilibrium pricing model. An optimization problem with two levels is being examined. The primary objective is to optimize the anticipated financial gain of the facility, taking into account the limitations imposed by storage resources and devices, as well as the adaptability of the facility. The lower level includes the market compensation pricing strategy, which aims to minimize the anticipated operating cost of the electrical and thermal generation units. While [2] focused on the energy management of grid-connected flexible energy hubs. A problem of minimizing the predicted operating cost is solved by applying it to the optimal power flow model of networks and the mathematical description of the utilization of energy hubs. The problem formulation has also taken into account uncertainties regarding the level of demand, the cost of electrical and heating energy, and the availability of renewable electricity.

The structure of a self-sufficient hybrid renewable energy system that can provide both electricity and thermal energy is studied in [3]. This system employs wind energy and bio-waste units using co-generation technology. Electric vehicles utilize smart charging to consume this energy. Compressed air energy storage is employed to equalize the production and usage of electricity, enhancing efficiency and diminishing cost. Thermal energy storage serves as an interface between renewable energy resources and thermal demands. The aim is to reduce the annual costs of the capital and maintenance of energy sources, storage facilities, and electronic power converters while taking into account the functioning of renewable sources, storage, and electric vehicles. For example, to analyze the energy management of a smart distribution network that incorporates hydrogen storage and renewable sources [4]. The objective is to assess the economic, operational, flexibility, and reliability features of the distribution system operator. The aim of this function is to minimize operational costs, enhance grid flexibility, and increase reliability. Stochastic scenario-based optimization is employed to represent uncertainties, such as variations in load, parameters associated with renewable resources, fluctuations in energy prices, and the availability of equipment.

To evaluate the opportunities of improving energy efficiency in energy centers, where they perform as aggregators and coordinators of multiple assets and storage systems, described in [5]. By effectively managing these resources and employing an appropriate energy management system, centers can anticipate achieving financial advantages in both the energy markets and auxiliary services. The goal is to optimize the overall profitability of the energy and reserve markets by maximizing the complete profile of the centers. The problem has been limited by the requirements of optimal power flow in gas, electricity, and thermal networks, as well as reserve limits and restrictions of the facilities, which encompass co-generation models, renewable energy resources, electrical/thermal storage, electric vehicle charging stations, and boilers.

The energy management of a virtual power plant consisting of a wind farm, energy storage technologies, and a demand response program is analyzed in [6]. The approach is executed at the electrical transmission level and involves interaction between virtual power plant in the daily energy and reserve markets. The goal is to synchronize the revenue of the Virtual Power Plants with the operational expenses of the power-generating units. The objective function is constrained by network constraints, reservation requirements for both upstream and downstream, and limitations particular to virtual power plant. A technique of hybrid stochastic-robust programming is utilized based in stochastic programming to address the uncertainty of day-ahead market prices, whereas robust optimization, which is based on bounded uncertainties, simulates uncertainties that are associated with wind farm load and power.

However, in [7] propose a two-level multi-objective model for evaluating the flexibility of a microgrid through the utilization of a flexible energy management system. A number of options are being considered: renewable resources, a demand response program, an energy storage system, and an integrated electric spring unit with electric vehicle parking. At the highest level, the model seeks to maximize the predicted benefit derived from flexible resources, while adhering to the limitations imposed by flexibility restrictions. At the lowest level, the objective functions aim to minimize the energy cost of the microgrid and the deviation in voltage, employing the Pareto optimization technique. Linearized AC optimum power flow constraints, limitations on renewable resources and flexibility, and limitations on microgrid adaptability all limit the performance of these functions.

On the other hand, in [8] suggest a model for flexible power management in a grid-connected microgrid that incorporates renewable energy sources and flexibility sources. This consists of a novel integrated electric spring unit that includes electric vehicle parking and a demand response program that operates based on incentives. The model is expressed as an optimization problem that aims to minimize the discrepancy between the anticipated energy cost and the anticipated advantage of flexibility sources. This is done while considering the limitations of AC optimal power flow flexibility, renewable energy sources, energy storage, and microgrids. Stochastic programming is employed to represent uncertainty in charging demand, energy prices, maximum renewable energy source generation capacity, and electric vehicle attributes.

The literature in [9] introduced Battery Energy Storage Systems (BESS) into the current definitions and classification of stability problems in power systems. This addition contributes to illustrating the advancements in storage devices and the growing importance that they have in modern energy systems. Therefore, by harnessing the potential of electrochemical storage, it may be employed to improve network conditions through its application and development. Ancillary services, congestion relief of the T&D, and voltage regulation are problems that storage can solve in times of economic crisis. Also, instability problems can be resolved using storage if used strategically, considering it as a power reserve since it is a reasonable option. The authors in [10] have detected problems in electrical networks, and it has founded possibilities to incorporate storage to provide virtual inertia and serve as backup during emergencies [9]. Due to this, strategic planning can take into account distributed or concentrated options. The integration of energy storage into distribution and transmission systems is a widely studied and analyzed alternative, as noted in references [11], [12], [13].

The impact of a solar and wind power development plan in Thailand, considering frequency response for system reliability and security, is analyzed in [14]. The integration of electrochemical and water storage in transitioning Saudi Arabia to a 100% renewable energy power system, highlighting the need for power system flexibility, is considered in [15]. The Central American region has identified the potential for employing PV and wind generation along with storage [16]. Similarly, studies conducted in Honduras and El Salvador, as cited in [17], [18], [19], have explored the use of storage for managing the power system's demand and enhancing voltage regulation. These analyses necessitate an examination of power flows, battery degradation, and ancillary services as frequency regulation, seasonal storage, and power reserves are discussed in [16], [17], [19], [20]. Storage can provide ancillary services, congestion relief of the T&D, voltage regulation, and allow grid investment deferral [21], [22], [23].

The authors in [24], [25] evaluate the potential of BESS to address voltage and frequency stability challenges in vulnerable power grids. The implementation of a binary gray wolf optimization technique has been used for identifying the most advantageous locations and sizing of the BESS, with the aim of improving the stability of voltage and frequency in a vulnerable network. Authors in [26] developed a modeling concept to maintain system stability amid the integration of various energy resources, increased demand, and decarbonization patterns. The authors assess the regional security of China's integrated energy system with renewable, identifying five risk points [27] in resources, generation, transmission, marketing, and consumption. Despite the fact that the power grid connections in closer proximity to one another exhibit greater responsiveness in addressing potential risks, there remains a discrepancy among various regions, which poses a significant energy risk for China.

Storage use with distributed generation is an option to mitigate the effects previously mentioned and thus improve stability, reliability, and loadability, among others. However, it should take into account the costs and benefits of grid power. Furthermore, integrating distributed storage can bring environmental benefits, mainly because it can remove non-renewable generation (of transmission) and replace it with PV and wind generation if it is managed with storage.

Storage distributed penetration could increase the distribution generation if used with PV generation [13], [28], [29], [30], [31]. There will be a change from a concentrated generation to a distributed generation with BESS in the future. This option may benefit the distribution system by improving power demand, increasing available power capacity, reserve, frequency regulation, energy availability in black starts, and load management.

Benefits in power transmission comprise load management, line congestion and investment deferral (planning), voltage stability and regulation, and power quality. One of the aspects to evaluate in energy storage for T&D networks is the provision of ancillary services such as frequency regulation, spinning and no-spinning reserves, black-start capability, and voltage regulation. Additionally, the enhancement of the T&D system's reliability and resilience, improvement of hosting capacity, provision of transmission VAR support, implementation of demand response, creation of microgrids, and management of power quality are also significant factors to consider.

In the context of power transmission and distribution electrical grids, incorporating energy storage is a crucial means of achieving short-term and long-term balance, as discussed in references [22], [32], [33], [34], [35], [36], [37]. Various factors, including ancillary services, wind and PV integration, grid investment deferral, congestion relief, and optimization, can be evaluated for this purpose. To incorporate new and emerging technologies, power systems must become increasingly flexible.

However, every action taken must substantially enhance the reliability of the system to meet the growing power demand and consumer requirements, as discussed in reference [38]. Actions such as increasing transmission capacities, enhancing system inertia, and integrating flexible generation have been adopted, as noted in reference [39]. Therefore, the power system's capability under any condition, such as high penetration of intermittent resources like wind or solar, must be assessed. Energy storage tends to be a critical option to increase the power system's capacities and ensure its stability, as discussed in [40].

BESS is an emerging technology that has acquired significance in the field of storage, particularly due to the growing use of electric vehicles. It is becoming increasingly important in power systems. It is important to take into account that employing second-life batteries in ESS offers an opportunity to utilize retired batteries that are going to be dumped by electric vehicles [41]. Hence, this article is focused on the widespread utilization of BESS in electrical grids. BESS has been used in such a situation to improve the stability of a power system.

An inevitable disadvantage is going to be the final disposal of the residual garbage generated by BESS [29], [37]. Nevertheless, these technologies can be used in power system applications [33], [35], [42]. Electrochemical storage technology is a potential and cost-effective technology that has significant potential for growth in the future, as previously mentioned. Table 1 shows multiple considerations associated with costs, allocations and sizing, evaluation of the power grid, reliability, flexibility, and management of demand and generation among other that can be implemented by ESS and offer solutions for multiple issues related to power grids.

**Table 1:** Studies Related to ESS.

ESS Applications	Issues	Paper
Cost Analysis & Optimal Dispatch	Life Cycle & Capital Cost, Grid Investment Deferral, Power Planning Allocation & Sizing Optimization	[1], [2], [3], [4], [5], [8], [12], [41], [43], [44], [45], [46], [47]
Power System Studies & Analysis	Stability, Power Quality, Spinning Reserve, Virtual Inertia Voltage Support and MVAR, electric energy time shift, Frequency Regulation, Renewable capacity firming Renewable energy time shift, Transmission congestion relief	[5], [6], [7], [9], [10], [25], [31], [48], [49], [50], [51], [52], [53], [54], [55], [56]
Operational Security & Reliability	Reliability & Resilience Enhancement, Ancillary Services, Power Quality, Voltage Regulation, Increasing Flexibility, Mitigating Transient Stability, Black-Start Capacity, Decarbonization	[11], [12], [13], [16], [17], [19], [20], [33], [36], [57], [58], [59], [60], [61], [62], [63]
Demand & Generation Management	Peak Load shaving, Load following, Increasing Available Power Capacity, Reserve, Load, Spinning and No Spinning Reserves, Improving renewable hosting capacity	[13], [17], [28], [29], [30], [31], [40], [64], [65], [66], [67], [68], [69], [70]

**Figure 1:**
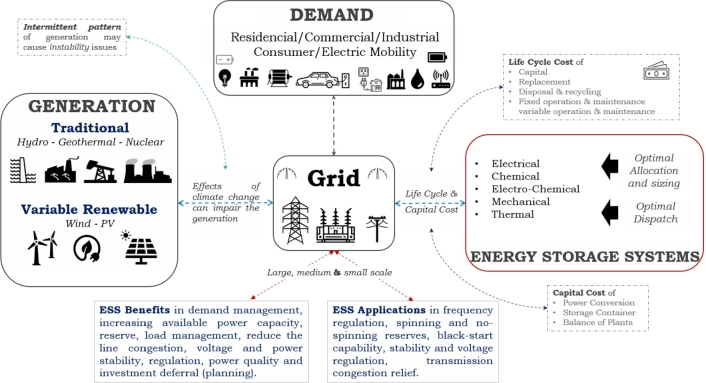
Graphical Abstract on main contributions.

Nowadays, there are several constraints impeding the expansion of electrical networks, resulting in deferral investments in the future, particularly in electrical infrastructure such as transmission and distribution. T&D can have issues such as power losses, constraints on power transfers, and problems regarding voltage regulation, as illustrated in Fig. 1. This figure additionally provides a graphical abstract and description of the main contributions, applications, and benefits that BESS may offer to power systems according to literature of the Table 1.

This article offers an approach for improving the flexibility of transfer capabilities between areas by utilizing BESS. The objective is to ensure the resilience, reliability, and stability of the system. In order to solve this problem, a method is used to find the BESS allocation by studying the stability and power flows of weak electrical grids.

The approach utilizes setting the BESS allocation in order to ensure that the system achieves its operational safety requirements. The main advantage of storage is its ability to decrease the risks of energy generation and demand imbalances, thus minimizing instability issues. It accomplishes this by supplying the required energy to maintain balancing and, if required, providing support in virtual inertia.

The paper is structured as follows: Section 2 describes the Material and Methods. The Results and Discussions are presented in Section 3. Finally, the Conclusions and future work are shown in Section 4.

## 2 Materials and methods

This section describes an approach to evaluating the addition of electrochemical storage in networks that are vulnerable to failure or disturbance. The goal of including BESS is to increase the virtual inertia capabilities of the network and offer support during critical conditions, thereby increasing the flexibility of the system. Furthermore, a solution has been offered for a particular event that took place within the Central American Regional Electrical System. The suggested approach entails incorporating storage to resolve a regional issue associated with vulnerable power grid topology.

**Figure 2:**
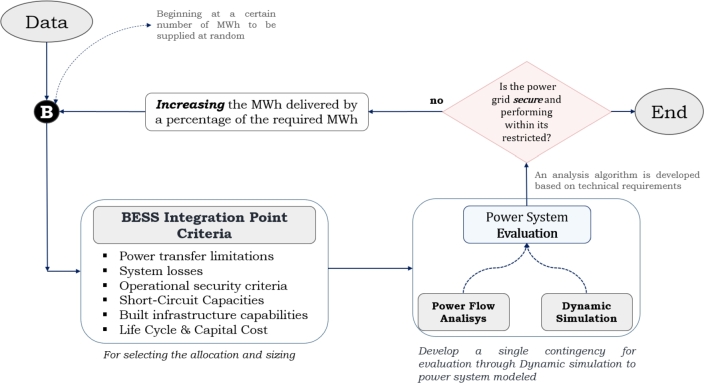
Flowchart outlining the systematic process to increase the flexibility of power transfers.

This approach utilizes a strategy for allocation and identifies the appropriate size of BESS in order to evaluate its viability for inclusion in a power grid. Fig. 2 illustrates a flowchart outlining the systematic process to increase the flexibility of power transfers using BESS. This approach operates as a sequential process in which the best places and sizes within a power system are chosen based on particular requirements.

Whenever evaluating a system, it is important to take into consideration multiple variables, including power transfer limitations, system losses, operational security standards, short-circuit capacities, built infrastructure capabilities, and life cycle and capital costs. Nevertheless, the possibility of including other criteria remains possible, as it will be contingent upon the features of the electrical grid under evaluation.

The suggested approach involves performing a simulation in the electrical system in order to evaluate the impacts of variations on energy flows and the stability of the energy. Through an analysis process, it can be determined whether these aspects within the simulation comply with the established operational security requirements. In order to achieve this, it is necessary to develop a strategy for analysis that incorporates all the aforementioned items.

If the operational safety, stability, or any other specified conditions are not satisfied, the process must be repeated by incrementing the MWh capabilities in the BESS. Such takes place in order to determine a point where the desired value is achieved not only in terms of capacity but also in considerations of the location and dimensions of the BESS. In the following section, a comprehensive depiction of the power system under evaluation and its application of the suggested approach can be detailed.

### 2.1 Power system analyzed

**Figure 3:**
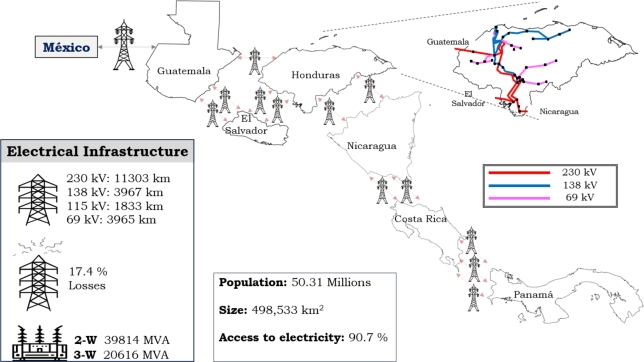
Regional Electrical System features based on [71], [72].

Guatemala, Honduras, El Salvador, Nicaragua, Costa Rica, and Panama comprise what is known as Central America. This region has a territorial extension of approximately 498 533 $km^{2}$. According to the Economic Commission for Latin America and the Caribbean (ECLAC) there are close to 50.31 million inhabitants [71], [72] as shown in Fig. 3 and the population of the region has access to electricity of 90.7%.

The Central American Electric System shows a diverse mix of electricity generation resources, where a large part of the electricity generated is from non-renewable energy. Transmission Power Grid mainly uses voltages of 230 kV, 138 kV, 115 kV and 69 kV, with losses of 17.4%. The countries of the region are linked through a 1,789.23 km-long regional transmission line at 230 kV. In 2020, the power demand reached 8,811.5 MW and the electricity production amounted to 51,522.2 GWh.

The Central American Power System was modeled and simulated in order to examine a significant disturbance previously described. Utilizing data from a regional power company (according to [72], [74]), a model was created for the event that took place on *June 9th, 2021*, and it recreated the same disturbance conditions according to the technical report of the regional electricity utility [73]. The Central American Power System was modeled and analyzed using the Electrical Transient Analyzer Program (ETAP®).

**Figure 4:**
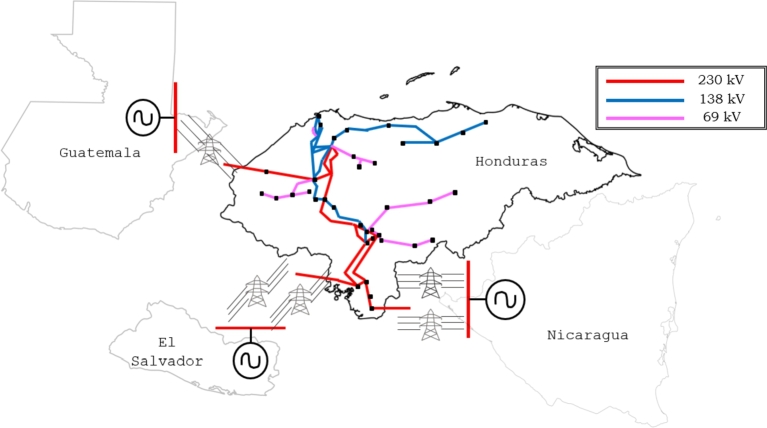
Power System Equivalent modeled.

Table 2 shows the features of the regional power system simulated and Fig. 3 shows the power transmission in the disturbance zone. A profile of demand and generation behavior was established for the conditions on June 9th, 2021, when the power demand in Honduras reached 1,563 MW. The power grid modeled includes renewable (hydro, wind, and PV) and non-renewable generation. The North Block (Mexico-Guatemala-El Salvador) is interconnected with Honduras and Nicaragua, which have elevated transmission losses [72] and the three interconnections among Mexico-Guatemala, El Salvador, and Nicaragua were modeled as *dynamic equivalents*, as shown in Fig. 4.

**Table 2:** Study Case Modeled and Simulated using ETAP based on [73], [74].

Description	Element Simulated	Capacities
Buses	193	230/138/34.5 kV
Transmission Lines	119	—
Generators - Production	49	37,989 MWh
Power Transformer	133	2-3 Winding
Loads - Demand	66	1,563 MW
Interconnections	5	GUA-MEX, SAL and NIC

The region exhibits certain distinct challenges, particularly in relation to the significant losses experienced by its electrical networks [72]. The deferral of investments in electrical infrastructure through transmission lines does not grow with demand and generation. The regional transmission line (SIEPAC) was initially constructed with a capacity of 300 MW. Limits resulting from losses or inadequate infrastructure within the countries, however, restrict the operation of the transmission line.

**Figure 5:**
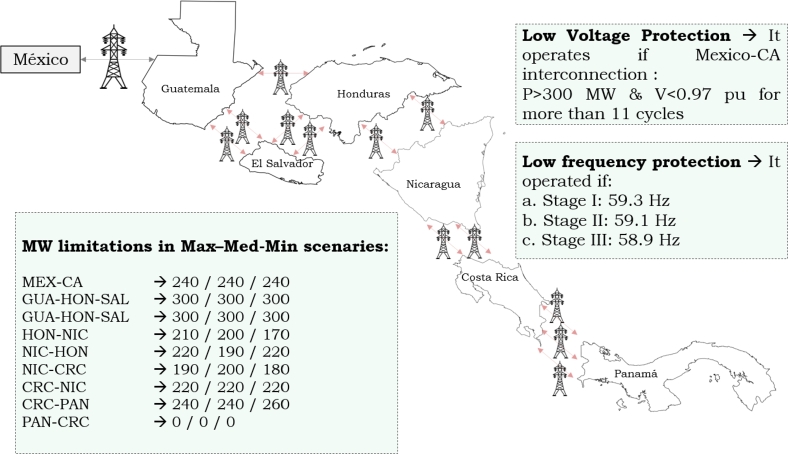
Power Transfer Limitations and Protections based on [72], [73].

Hence, the restrictions on power transfers among the countries of the region can be observed, as depicted in Fig. 5. It is evident that the absence of restrictions within the countries comprising the northern block (Guatemala, Honduras, and El Salvador) can be attributed to the electrical configuration of the region. Additionally, the close proximity to a robust node (Mexico) significantly enhances the stability of this area. Nevertheless, the southern block, including Nicaragua, Costa Rica, and Panama, exhibits a radial structure with interconnections that display restrictions during various times of the day, featuring minimum, medium, and maximum levels.

The consideration of Panama's lack of connection with South America holds significant importance. The protection of the system is achieved through the maintenance of the balance between demand and generation; failure to meet these requirements could potentially result in operational issues. To ensure the operational security of the system and minimize the potential for generation loss, a protective strategy utilizing low-frequency protection has been deployed. This technique functions through three discrete stages, as depicted in Fig. 5 disconnecting several loads in substations.

Problems in transmission and generation capacities have affected power transfer and operation among countries, notwithstanding that the use of Supplementary Control Schemes (SCS) has been necessary to solve regional electrical problems. The adjustments made to the SCS are depicted in [72], with the aim of preventing issues such as instability, power imbalance, and other potential risks that may arise during the operation of the regional system. It should be noted that the power capacities among countries have been constrained due to high losses in their respective electrical networks and insufficient transmission infrastructure. Although the regional transmission line has a capacity of 300 MW, it has been limited in some countries.

**Figure 6:**
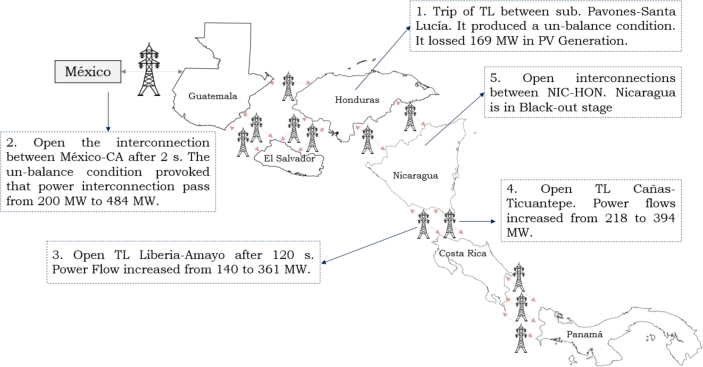
Regional Power System Black-out Sequence based on [72], [73].

If voltage and power levels (Mexico Interconnection) become unsafe during operation, the SCS (protection mechanism) is programmed to open this interconnection after 11 cycles under certain operating conditions. Some disturbances can affect the system frequency, and it is necessary to disconnect the load or generation to maintain the power balance and stabilize the frequency at 60 Hz. These factors have led to the identification of operational risks between the Mexico interconnection and the Central American Power System, where the maximum capacity power transfer is limited to 300 MW with a voltage that remains above 0.97 p.u. as shown in Fig. 6. Some risk conditions due to the saturation of transmission lines or excess renewable generation may arise and cause power imbalances in the system.

On June 9th, 2021, there was a partial blackout in the Central American area. This disturbance resulted in a significant decrease in frequency within the Central American Electrical System and caused an electrical imbalance, leading to a blackout in the Nicaraguan Electrical System. The interconnection voltage levels plummeted to $V_{int}$ ≤ 0.97 pu when the power level was at 469 MW.

When $P_{int}$ was greater than 300 MW and $V_{int}$ was less than 0.97 pu for at least 11 cycles, a low-frequency response was seen. This meant that the SCS was activated, which caused the frequency drop. This led to the activation of the protection mechanism, which disconnected the Mexico Interconnection. The transmission network exhibits significant loss conditions among areas comprising the Northern Block (Mexico-Guatemala-El Salvador) and the Southern Block (Costa Rica-Panama) due to the presence of considerable transmission losses within the Honduras-Nicaragua segment.

The activation of SCS was to prevent frequency from decreasing to 59.3 Hz since regional protection recognizes it and the power breaker removes loads until supply and demand balance is attained. The disturbance induced a decrease in frequency to 58.75 Hz, thereby activating low-frequency protection, while the power deficit was 1,321.0 MW in the region.

The power imbalance in the Central American Power System was caused by the events depicted in Fig. 6, which are as follows:

1. The disconnection of a transmission line within the Honduras Power Grid resulted in a high power transmission, leading to an unbalanced condition within the Regional Power System. This ultimately caused a loss of 169 MW in PV Generation.
2. The power imbalance led to a shift in power flow (Mexico interconnection) from 200 to 484 MW. Subsequently, the power breaker opened the connection between Mexico and Central America after 2 seconds.
3. Following the opening of the Mexico interconnection, the regional frequency dropped to low levels. Upon reaching this level, the low frequency protections were activated, ultimately resulting in the third stage of protection being triggered.
4. Subsequent the incident, the power flow between Costa Rica and Nicaragua (interconnection 1) increased from 140 to 361 MW, resulting in the opening of the power breaker.
5. The power flow between Costa Rica and Nicaragua (interconnection 2) rose from 218 to 394 MW, leading to the opening of the power breaker.
6. The power flow between the interconnection of Honduras and Nicaragua increased, resulting in the opening of the two interconnections by the power breaker.
7. Nicaragua experienced a state of severe blackout.

### 2.2 June 9th, 2021 blackout simulated

**Figure 7:**
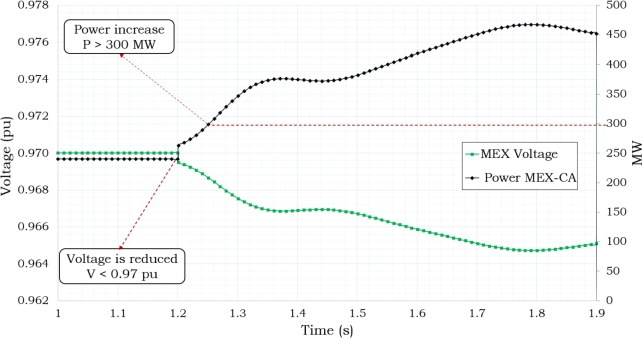
Power and Voltage Behavior in Mexico's Interconnection based on [72], [74].

**Figure 8:**
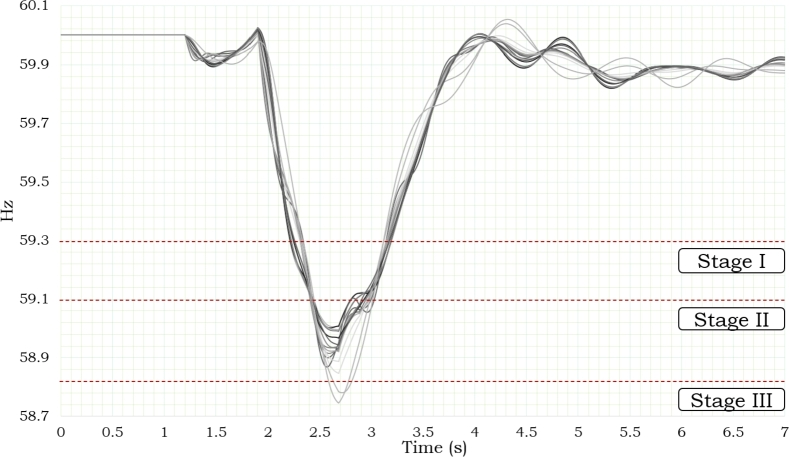
Frequency in Several Nodes of Power System based on [72], [74].

The interconnection voltage levels dropped to $V_{int}$ ≤ 0.97 pu when the power level was at 469 MW, as shown in Fig. 7. Fig. 8 shows the frequency response that was seen during 7 seconds of simulation of the disturbance. As shown in this Figure, the Supplementary Control Scheme activated the protection when both $P_{int}$ ≥ 300 MW and $V_{int}$ ≤ 0.97 p.u. conditions were present at the same time for T ≥ 11 cycles.

Later, the halt of connectivity between Mexico and Central America resulted instability conditions, which resulted in a reduction in the frequency. Consequently, the activation of low-frequency protective mechanisms within the regional system resulted in disconnecting several loads in the power grid. Fig. 8 shows the various stages of load disconnection for low-frequency protection, and the stages are adjusted at three distinct frequencies (59.3, 59.1, and 58.9 Hz) as depicted in Fig. 5.

The proposal for incorporating BESS as an approach for improving system stability [16] stems from the need to introduce Virtual Inertia in response to the vulnerability of the Central American system to certain disturbances.

Due to the concentration of hydroelectric power in the southern region, the implementation of virtual inertia in the northern region might prevent the activation of Complementary Control Schemes. It has an opportunity to enhance energy flexibility and increase the capacity for energy transmission among various areas.

### 2.3 Increasing power flexibility methodology

An algorithm is put forth to establish the integration of electrochemical storage within the electrical power grid, with the main objective of reducing the adverse impacts arising from large disturbances. Within this context, several integration points (*i*,*k*) are identified throughout the system, representing electrical substations (buses) operating at voltages of 230 and/or 138 kV.

Algorithm 1 takes into careful consideration the conditions and security criteria delineated in the power system [72] and the proposed approach involves verifying that $P_{int}$ ≤300 MW and $V_{int}$ ≥0.97 pu are maintained within an interval of 11 cycles, without surpassing these thresholds, thereby preventing the disconnection of the interconnection with Mexico.

**Algorithm 1:**
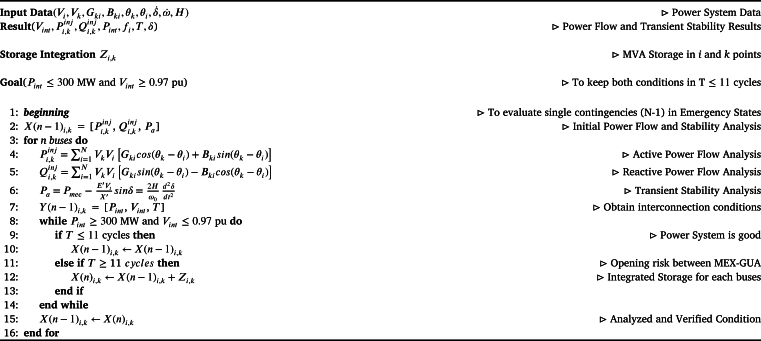
Defining BESS Integration Point in Central America Power Grid.

To achieve this, power flow and transient stability analyses are being developed to assess voltage levels, power flow magnitudes, and frequency values within the interconnection. This comprehensive evaluation aids in identifying the most favorable siting that meets the operational security established. The methodology's input data, results, and requirements are described below to achieve the stated objective.

The methodology's input data, results, and requirements are described below to achieve the stated objective.

• **Input Data:**
  Input data are the bus voltage ($V_{i}$, $V_{k}$), admittance of the transmission lines, transformers, generators, load ($G_{ki}$, $B_{ki}$, $\theta_{k}$, $\theta_{i}$) and dynamic data ($\overset{˙}{\delta }$, $\overset{˙}{\omega }$, ${\overset{˙}{E}}_{d}^{\prime \prime }$, ${\overset{˙}{E}}_{q}^{\prime \prime }$, ${\overset{˙}{E}}_{d}^{\prime }$, ${\overset{˙}{E}}_{q}^{\prime }$, H) among others.
• **Results:** The results provide the voltage and power flows in Mexico interconnection ($V_{int}$, $P_{int}$) and between and inside the countries ($P_{k}^{inj}$, $Q_{k}^{inj}$). Similarly, frequency ($f_{i}$) and stability angle (*δ*) are determined from the transient stability study. *T*, it is the time when $P_{int}$ ≥ 300 MW and $V_{int}$ ≤ 0.97 pu are present simultaneously. So, the convenient point of storage is defined in order to maintain the power balance and operating conditions of the system defined.
• **Goal:** To avoid the disconnection of the interconnection with Mexico, it is necessary to maintain the power in $P_{int}$ ≤ 300 MW and the voltage in $V_{int}$ ≥ 0.97 pu.
• **Beginning:** The beginning of the process contemplates the input data to analyze the initial information. The simulation performs the analyze to determine the operating conditions of the grid. Finally, it establishes the best storage point to locate it inside the power system.
• **Operating conditions estimator:**
  Power flows are determined to keep them within the current values. Power ($P_{k}^{inj}$, $Q_{k}^{inj}$) and Voltages ($V_{i}$, $V_{k}$) are calculated. Similarly, the frequency in each of the countries ($f_{i}$) and the Mexico Interconnection ($P_{int}$, $V_{int}$).
• **Storage endpoint estimator:** When *Y* has simultaneous conditions (of $P_{int}$ ≥ 300 MW and $V_{int}$ ≤ 0.97 pu), time (T) begins to count. If T ≤ 11 cycles, the power system does not have risk and the power breaker between Mexico and Guatemala is maintained closed. Nevertheless, if T ≥ 11 cycles, the power system has a instability risk because the power breaker between Mexico and Guatemala is open. In this case, $Z_{i,k}$ models the storage integration at the 230 and 138 kV nodes until the condition *T* ≤ 11 cycles and $P_{int}$ ≤ 300 MW and $V_{int}$ ≥ 0.97 pu is reached.
• **End:** This is the end of the definition stage of Storage integration point into the power grid. The model can establish the optimal point to integrate the concentrated storage into the system, reducing its instability risk.

The Algorithm 1 outlines a methodology that can be used to plan a network by initially identifying potential risks to the system and then making cost-effective decisions about the strategic placement of storage throughout the network. As a result, this methodology is suitable for the current system but can also be modified to accommodate different network scenarios.

## 3 Results and discussions

The algorithm presented in the preceding section offers a method for integrating electrochemical storage. According to the authors' discussion in [16], [17], this method offers a solution for electrical networks that are susceptible to changes in network topology or system operating conditions. Two separate studies were done on the integration point that was looked at to find ways to fix problems. The first study looked at how storage could be used in the 230 kV transmission system, and the second study focused on the 138 kV system, as shown in Table 3.

**Table 3:** Cases Analyzed and Capacities for BESS.

Case	Mode	230kV	138 kV	Figure
1	Distributed	80 MW	80 MW	8 9(a)
2	Distributed	80 MW ⁎	80 MW	9(a)
3	Concentrated	80 MW ⁎	80 MW	9(b)
4	Concentrated	80 MW	80 MW ⁎	9(b)

⁎ Power delivered in emergency cases

In order to solve the issue of restoring the energy that was lost in the investigated event, it has been suggested to employ a BESS (Battery Energy Storage System) with a capacity of 1,060 MW h/160 MW and a virtual inertia of H=6s. Storage has been considered from both a concentrated and distributed approach. Concentrated refers to the integration of large power blocks inside one node, whereas distributed involves the incorporation of storage into smaller units that are shared among several nodes.

This study includes the evaluation of two nodes in the concentrated case and eight nodes in the distributed case. Each single node inside the system represents a power substation, and storage was modeled, simulated, and integrated into power substations. The addition of small power blocks and avoiding the possibility of system instability conditions were important considerations in the selection of 20 MW modules.

**Figure 9:**
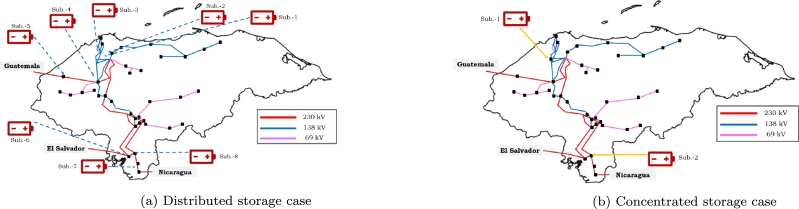
Selected points where storage is installed according to Algorithm 1.

In order to mitigate the risk of disconnection in the interconnection between Mexico and the regional power system, this methodology emphasizes the improvement of flexibility in power transfers. The disturbance resulted in an energy imbalance within the regional system, consequently requiring 167 MW. The application of the method in the context of the regional electrical system has resulted in the identification of two potential proposals, as illustrated in Fig. 9. Consequently, the following options will be given for the evaluated BESS capacity cases, as shown in Table 4.

**Table 4:** Cases Analyzed and Capacities for BESS.

Case	Mode	MW h	MW	Units
1	Distributed	132.5	20	8
2	Distributed	132.5	20	8
3	Concentrated	530	80	2
4	Concentrated	530	80	2

This figure shows the most favorable allocation (each allocation represents a power substation in the electrical grid) where the system operated satisfactorily, taking into account the voltage, power, and frequency requirements to prevent disconnection of interconnection. The first solution, presented in Fig. 9(a), revolves around the implementation of distributed BESS across eight distinct allocations within the power system.

On the other hand, Fig. 9(b) showcases an alternative approach involving storage implementation at two specific allocations within the system (concentrated). The evaluation focuses on the possibility of utilizing 50% of the BESS in concentrated scenarios while reserving the remaining portion for emergency situations. The simulation took place in the Honduran power system, particularly in an area of the electricity grid that experiences major losses which is where the disturbance occurred.

### 3.1 Case 1

**Figure 10:**
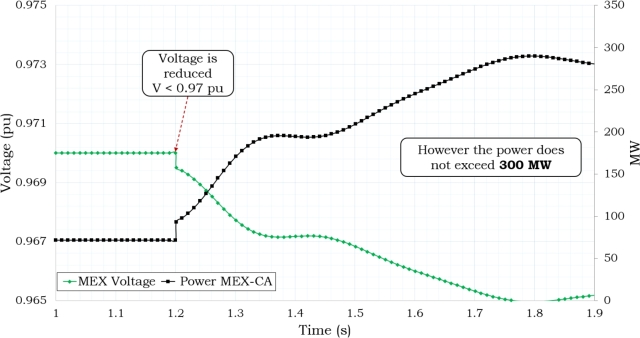
Delivering Storage of 160 MW.

In accordance with Table 3, Table 4, 160 MW of power is distributed in eight sites of the power system (refer to Fig. 10) in units of 20 MW each. In the present case, it is pointed out that distributed dispatching, as depicted in Fig. 9(a), signifies an appropriate solution to maintenance the stability of the interconnection with Mexico and Central America, so indicating favorable conditions for its utilization. Compensation takes place on both the 230 kV and 138 kV sides in order to assure the power balance in loads. The significance of the issue is in its capacity to effectively manage the demand behavior of the system, hence reducing the possibility of significant power transfers among regions.

### 3.2 Case 2

**Figure 11:**
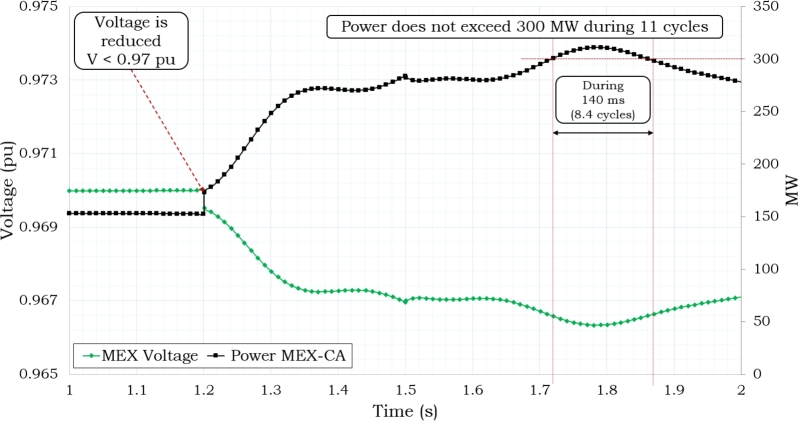
Delivering Storage of 80 MW.

Based on the data shown in Table 3, Table 4, it can be observed that the power system is designed to distribute a total of 80 MW of electricity among four sites, as depicted in Fig. 9(a). Each site receives an allocation of 20 MW. Additionally, there is an additional 80 MW of power reserved for emergency situations. The installation of storage on the 230 kV side remains in place for the purpose of addressing situations of emergency. A satisfactory result can also be obtained by compensating the system in a distributed way while using half of the storage for power delivery and the other half as a backup, as shown in Fig. 11. In this scenario, the 230 kV-side are supplied with a constant power delivery of 80 MW during the system's peak demand hours, while the backup storage is located on the 138 kV-side. This approach reduces the risk of disconnection from Mexico as the voltage and power levels are kept within the required ranges. However, it should be noted that the condition of $P_{int}$ ≥ 300 MW and $V_{int}$ ≤ 0.97 pu occurs for 8.4 cycles (140 ms), although this behavior is still acceptable.

### 3.3 Case 3

In accordance with the information shown in Table 3, Table 4, the power system has been designed to dispatch power to a single node, as shown illustrated in Fig. 9(b). In this analysis, the dispatch of 80 MW of storage can be seen on the 138 kV side, while simultaneously assuring the presence of 80 MW on the 230 kV side as a precautionary measure for emergency situations.

**Figure 12:**
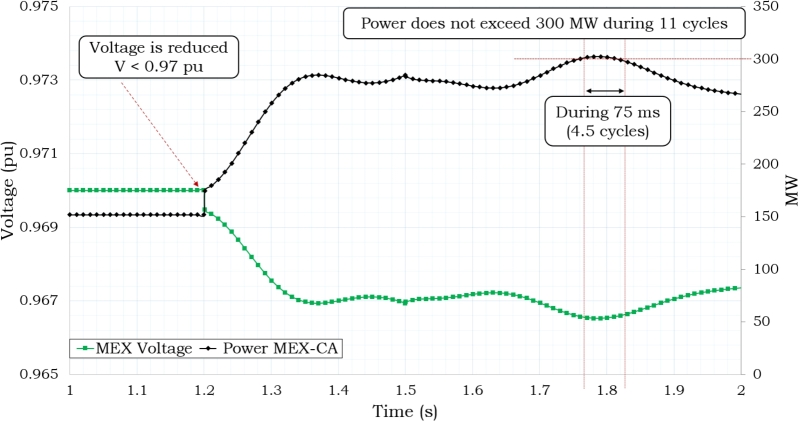
Delivering 80 MW in 138 kV-side.

This condition poses no risk to the regional power system, and the condition of $P_{int}$ ≥ 300 MW and $V_{int}$ ≤ 0.97 pu occurs for 4.5 cycles (75 ms) as shown in Fig. 12. The achievement of stability conditions takes place through the interconnection, consequently minimizing the potentiality of initiating the opening of the interconnection with Mexico.

### 3.4 Case 4

**Figure 13:**
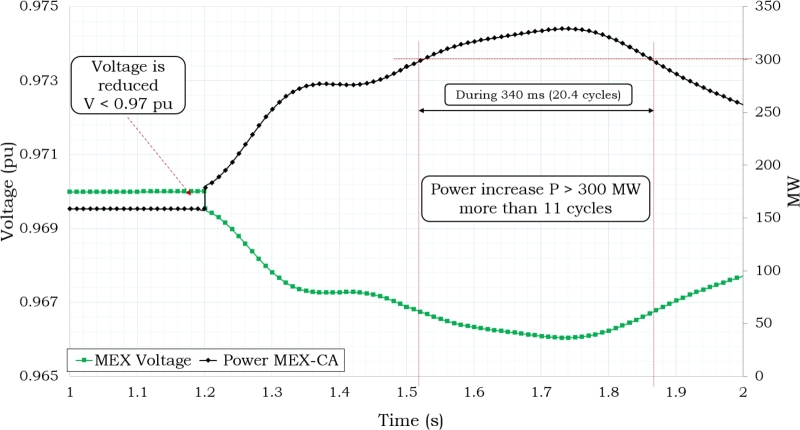
Delivering 80 MW in 230 kV-side.

Case 4 examines the scenario wherein power dispatch operates on the 230 kV side while the 138 kV side is reserved for situations of emergency, as shown by the data presented in Table 3, Table 4 and Fig. 9(b). If the power is dispatched from the 230 kV-side, as shown in Fig. 13, the power and voltage interconnection do not reach satisfactory operating conditions, resulting in a state of risk of disconnection due to the condition of $P_{int}$ ≥ 300 MW and $V_{int}$ ≤ 0.97 pu, which persists for more than 11 cycles. In this particular scenario, it is quite likely, given specific operational circumstances, that the system may encounter issues related to instability. It has been observed that direct compensating in the load is necessary due to the enormous losses experienced in the transmission networks, which impose restrictions on power transfer.

### 3.5 Frequency and demand respond analysis

**Figure 14:**
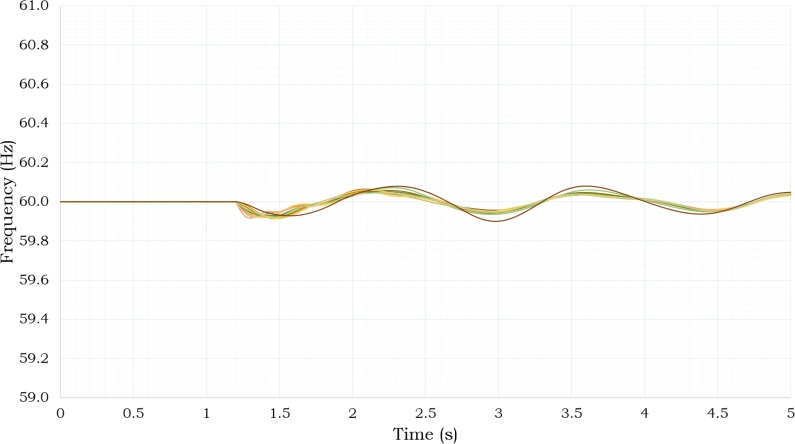
Frequency Behavior.

**Figure 15:**
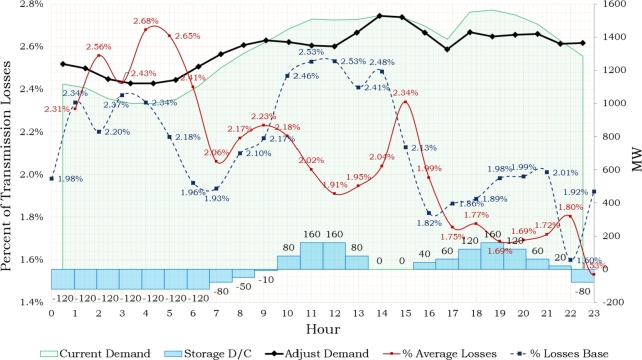
Demand Respond, losses and Storage Management.

All cases analyzed exhibit a stable frequency behavior for the regional electrical system, as demonstrated in Fig. 14. During the simulations, the compensation resulted in a frequency variation of ±0.1 Hz. Fig. 15 illustrates the demand respond. Green curve depicts the demand on the day of the event (24-hour analysis), while the black curve shows a modification of the demand behavior due to storage integration simulation. Charging and dispatching of storage have been implemented in order to maximize cost efficiency by doing the charging during minimal time demand, while ensuring timely dispatch during periods of high demand. Fig. 15 illustrates the variations in percentage losses over a 24-hour period. There is a distinct tendency towards increase over the diurnal period posterior to 6 am. The proposed storage management strategy indicates a reduction in losses, as seen by the red curve. Percent losses show a pattern of increase during nocturnal hours due to the charging stage of storage units.

## 4 Conclusions

A approach is suggested for incorporating BESS into networks that are vulnerable due to contingencies, deficiencies in generation capacity, or modifications to the topology, among other factors. The technique identified the hosting locations to enhance the operational security of the system by improving the network's flexibility.

Electrical grids may encounter technological issues that, as a result of inadequate planning or insufficient investments, can render them more susceptible and less dependable. Hence, effective strategic planning may guarantee that investments are done during the most advantageous periods, thereby reducing the necessity for more expensive alternatives. In order to tackle these difficulties, the integration of technologies like electrochemical storage might enhance the resilience of electrical networks, as explained in the article.

Adopting energy management strategies that make use of PV and wind power generation, which BESS can control in either distribution or transmission networks, is one potential option to think about. This alternative is a significant answer when dealing with electrical networks that are vulnerable to potential disturbances, and it exhibits significant potential for use in Central American networks, where it has been proven that there are vulnerabilities to instability.

According to the study, using criteria to figure out allocation and sizing at key points in sensitive systems can lead to more flexible transfers, which gets rid of any possible boundaries. The Central American electrical network, which has limitations in power transfers, is appropriate for the use of BESS. This is because during severe contingencies, such as the one analyzed, where the system frequency dropped to 58.75 Hz and the power transfer between Mexico and Central America exceeded 300 MW with voltage levels below 0.97 pu, the use of BESS can prevent these types of problems.

The proposed solution proposes using BESS in both concentrated and distributed manners. In the concentrated approach, eight potential allocations have been selected and listed for implementation, while in the distributed approach, two BESS units are used to supply power and manage the general imbalance of energy between generation and demand. The results demonstrate that the possibility of instability decreases as the power does not exceed a 300 MW increase by more than 11 study cycles for all scenarios. The proposed approach includes establishing a BESS with an installed capacity of 1,060 MW h/160 MW and a virtual inertia of H=6s.

Whenever power dispatch occurs on the 230 kV side and the 138 kV side is utilized only for situations of emergency, there is a possibility that the dispatch from this side may result in a power and voltage interconnection that does not meet the desired operating conditions. The condition of $P_{int}\geq 300$ MW and $V_{int}\leq 0.97$ pu induces an environment of risk for disconnection that lasts for more than 11 cycles. With these operating conditions, there is a much higher chance of instability, so it is suggested to directly compensate for the load (138 kV-side) because of the losses in transmission networks that make it hard for energy transfer between transmission lines.

Notwithstanding, due to the constant advancement of technology, it is necessary to take into account both technological and financial limitations. The ultimate disposal of the garbage constitutes an engineering challenge that must be taken into consideration, and evaluating the cost of acquisition is an important consideration in analyzing the economic viability of significant projects. However, the ESS option is outlined as a plausible short-term strategy for solving issues relative to deferral investments in electrical infrastructure. The research indicates positive prospects for the ESS, as it requires funding for developing short-term research in multiple fields such as engineering and integration, modeling and simulation, economic evaluation, safety and operation, technology and manufacturing, and the impact on electrical networks.

Energy planning should consider short, medium, and long-term perspectives, follow reliable criteria and established methods, and draw on experiences and knowledge gained from other sources. Integrating new energy alternatives (such as BESS) is crucial for enhancing the flexibility of power, voltage, and transfer capacities among countries.

## CRediT authorship contribution statement

**Gustavo Adolfo Gómez-Ramírez:** Writing – review & editing, Writing – original draft, Resources, Project administration, Methodology, Investigation, Formal analysis, Data curation, Conceptualization. **Luis García-Santander:** Writing – review & editing, Supervision, Conceptualization. **Markel Zubiaga Lazkano:** Writing – review & editing, Supervision, Conceptualization. **Carlos Meza:** Writing – review & editing, Supervision, Conceptualization.

## Declaration of Competing Interest

The authors declare that they have no known competing financial interests or personal relationships that could have appeared to influence the work reported in this paper.

## Data Availability

All data will be provided upon request.
